# Longer Poly(U) Stretches in the 3′UTR Are Essential for Replication of the Hepatitis C Virus Genotype 4a Clone in *in vitro* and *in vivo*

**DOI:** 10.3389/fmicb.2021.764816

**Published:** 2021-11-25

**Authors:** Asako Takagi, Yutaka Amako, Daisuke Yamane, Bouchra Kitab, Yuko Tokunaga, Ahmed El-Gohary, Michinori Kohara, Kyoko Tsukiyama-Kohara

**Affiliations:** ^1^Department of Microbiology and Cell Biology, Tokyo Metropolitan Institute of Medical Science, Tokyo, Japan; ^2^Joint Faculty of Veterinary Medicine, Transboundary Animal Diseases Centre, Kagoshima University, Kagoshima, Japan; ^3^Laboratory of Animal Hygiene, Joint Faculty of Veterinary Medicine, Kagoshima University, Kagoshima, Japan; ^4^Egypt-Japan University of Science and Technology, New-Borg El Arab City, Egypt; ^5^Faculty of Medicine, Suez Canal University, Ismailia, Egypt

**Keywords:** hepatitis C virus, genotype 4a, infectivity, humanized chimeric mouse, genomics

## Abstract

The 3′ untranslated region (UTR) of the hepatitis C virus (HCV) genome plays a significant role in replication including the poly(U) tract (You and Rice, 2008). Here we established an HCV clone that is infectious *in vitro* and *in vivo*, from an Egyptian patient with chronic HCV infection and hepatocellular carcinoma (HCC). First, we inoculated the patient plasma into a humanized chimeric mouse and passaged. We observed HCV genotype 4a propagation in the chimeric mouse sera at 1.7 × 10^7^ copies/mL after 6 weeks. Next, we cloned the entire HCV sequence from the HCV-infected chimeric mouse sera using RT-PCR, and 5′ and 3′ RACE methodologies. We obtained first a shorter clone (HCV-G4 KM short, GenBank: AB795432.1), which contained 9,545 nucleotides with 341 nucleotides of the 5′UTR and 177 nucleotides of the 3′UTR, and this was frequently obtained for unknown reasons. We also obtained a longer clone by dividing the HCV genome into three fragments and the poly (U) sequences. We obtained a longer 3′UTR sequence than that of the HCV-G4 KM short clone, which contained 9,617 nucleotides. This longer clone possessed a 3′-UTR of 249 nucleotides (HCV-G4 KM long, GenBank: AB795432.2), because of a 71-nucleotide longer poly (U) stretch. The HCV-G4-KM long clone, but not the HCV-G4-KM short clone, could establish infection in human hepatoma HuH-7 cells. HCV RNAs carrying a nanoluciferase (NL) reporter were also constructed and higher replication activity was observed with G4-KM long-NL *in vitro*. Next, both short and long RNAs were intra-hepatically injected into humanized chimeric mice. Viral propagation was only observed for the chimeric mouse injected with the HCV-G4 KM long RNA in the sera after 21 days (1.64 × 10^6^ copies/mL) and continued until 10 weeks post inoculation (wpi; 1.45–4.74 × 10^7^ copies/mL). Moreover, sequencing of the HCV genome in mouse sera at 6 wpi revealed the sequence of the HCV-G4-KM long clone. Thus, the *in vitro* and *in vivo* results of this study indicate that the sequence of the HCV-G4-KM long RNA is that of an infectious clone.

## Introduction

Hepatitis C virus (HCV) belongs to the *Flaviviridae* family and genus *Hepacivirus* and possesses a single-stranded RNA with positive polarity (Choo et al., 1989). Approximately, 58 million people are estimated to be currently infected with HCV (WHO, 2021). Based on the diversity of the HCV genome, it has been classified into seven genetically distinct genotypes (HCV 1–7) (Smith et al., 2014; Tsukiyama-Kohara and Kohara, 2017). A recent report suggested the existence of eight HCV genotypes (Borgia et al., 2018). HCV genotype 4 appears to be mainly found in Africa and the Middle East; in particular, HCV isolated in Egypt is mostly classified as genotype 4 (Iles et al., 2014). The characteristics of HCV genotype 4 have not been fully clarified, mostly because of the lack of an available infection system. An infectious clone of HCV genotype 4a has been previously developed *in vivo* (Strain ED43) (Gottwein et al., 2010), and was cloned from the plasma of chimpanzees inoculated with plasma from patients with chronic hepatitis C.

After the discovery of HCV (Choo et al., 1989; Kolykhalov et al., 1997), an efficient infection system was first established using the HCV genotype 2a clone, JFH-1 (Lindenbach et al., 2005; Wakita et al., 2005). The HCV JFH-1 clones can replicate both *in vitro* and *in vivo*. This HCV infection system has contributed to the development of direct-acting antivirals (DAAs), which target HCV protease and polymerase and efficiently suppress viral replication (Spengler, 2018).

To better understand the characteristics of HCV genotype 4a in patient plasma, we aimed to construct an infectious clone for HCV genotype 4a from chimeric mice with humanized livers (Mercer et al., 2001) inoculated with Egyptian chronic hepatitis C patient plasma. We first cloned the full-length sequence of the HCV genome from the sera of these chimeric mice. We obtained clones with a shorter 3′UTR (G4 KM short) and longer 3′UTR (G4 KM long). It has been previously reported that 3′UTR plays a significant role in HCV replication, and this is composed of a stem-loop structure, poly(U) stretch and 3′X region (Tanaka et al., 1995; You and Rice, 2008). The infectivity of the HCV genotype 4a clones was examined by transfecting of RNA into cell culture and chimeric mice with humanized livers.

## Materials and Methods

### Infection of Hepatitis C Virus Genotype 4a in Chimeric Mice With Humanized Liver

Chimeric mice with humanized livers were purchased from PhoenixBio Co., Ltd. They were intravenously infected with plasma from Egyptian patients (HCV-positive, multiple hepatic focal lesions, splenomegaly) containing 10^4^ copies of viral RNA (Katsume et al., 2013). After a few weeks, the mice were sacrificed, and sera were obtained for further experimentation. Isolation and quantitation of HCV-RNA were performed, as described previously (Takeuchi et al., 1999).

### Cloning of Hepatitis C Virus Genotype 4a cDNA and Construction of an Infectious Clone

**FIGURE 1:**
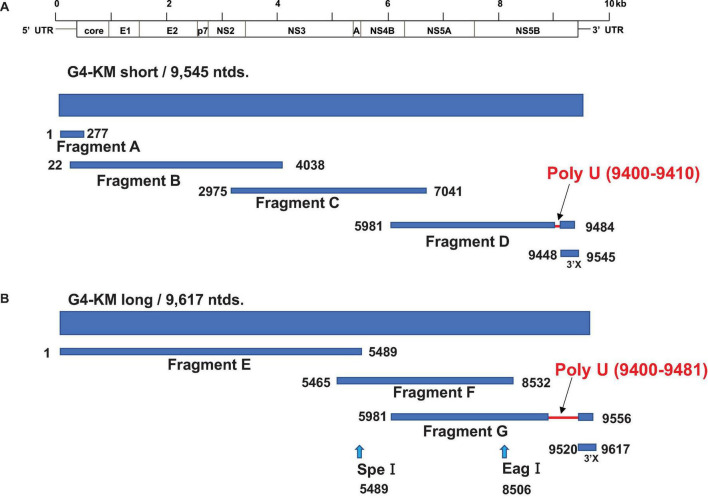
Strategy for HCV-G4 cloning. (A) Strategy for HCV-G4 KM short cloning. Fragment A, B, C, D and 3′X were amplified from cDNA which was reverse transcribed with RNA isolated from HCV-G4- infected chimeric mouse sera. HCV genes were amplified using primer sets of each fragment as described in Table 1. (B) Fragment E was generated from Fragment A, B, and C using restriction enzymes. We obtained Fragment G with a 71-nucleotide longer poly U sequence, which was used for the construction of HCV-G4 KM long.

Construction of the infectious HCV genotype 4a clone was performed as follows. First, RNA was extracted from the serum of a chimeric mouse using the acid guanidinium thiocyanate-phenol-chloroform extraction method, as described previously (Katsume et al., 2013) or SepaGene RV-R (Sanko Junyaku Co.). Then, cDNA was synthesized using Superscript III reverse transcriptase and amplified using PrimeSTAR GXL DNA polymerase (Takara Bio Co.) in fragment A–G and 3′X (Figure 1) with the primer sets described in Table 1. PCR conditions were as follows: 98°C for 5 min, 30 cycles of 98°C for 10 s, 60°C for 15 s, and 68°C for 1 min/kb, and finally 68°C for 7 min. Amplified DNA fragments were subcloned into a low-copy number vector, pSMART (Lucigen Co.). Construction of the infectious HCV genotype 4a clone was performed using restriction enzymes or an In-Fusion HD cloning kit (Clontech Co.).

**TABLE 1:** Primers used to clone HCV genotype 4a.

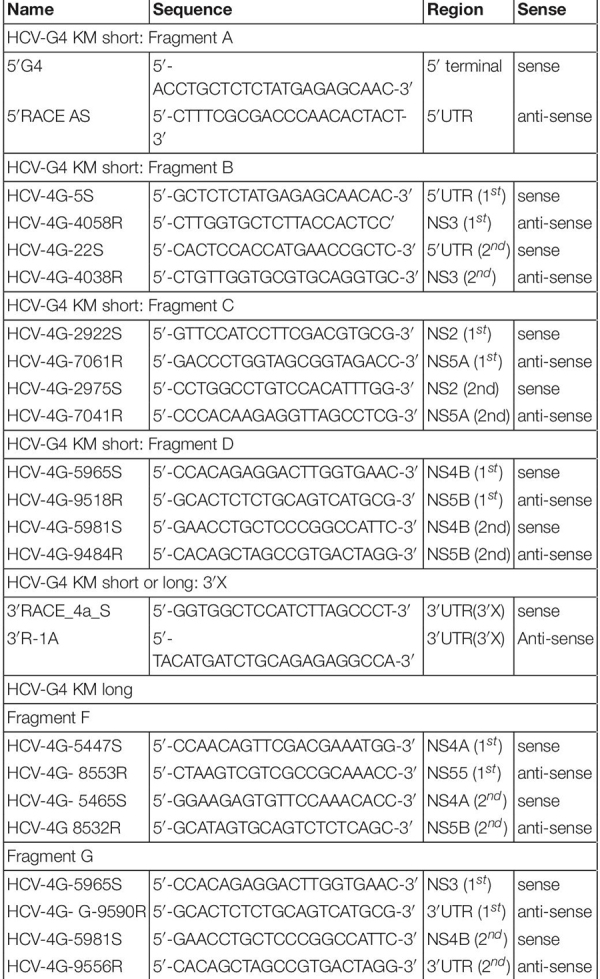

*Fragment E was generated from Fragment A, B and C using restriction enzymes.

To clone the 5′UTR, total RNA was extracted from chimeric mouse sera (10 mL) with ISOGEN-LS and treated with DNase, followed by cDNA synthesis using Superscript III reverse transcriptase (Thermo Fisher Scientific Co.) and a 5′RACE antisense primer (5′RACE System for amplification of cDNA ends, ver. 2, Invitrogen Co.). The target region was amplified after dCTP tailing, according to the manufacturer’s protocol. Cloning of the 3′UTR was performed using RNA extracted from chimeric mice; cDNA was synthesized using Superscript III reverse transcriptase and amplified, as reported previously (Tanaka et al., 1996; Tsuchihara et al., 1997).

### Confirmation of the Hepatitis C Virus cDNA Clone

Transcription of HCV-G4 KM short or HCV G4-KM long RNA was performed using the MEGA script T7 transcription kit (Thermo Fisher Scientific Co.) or T7 RiboMax Express kit (Promega Co.) and characterized by formaldehyde agarose gel electrophoresis. The transcribed RNAs were purified using NucAway spin columns (Thermo Fisher Scientific Co.) and phenol chloroform extraction. RNA size markers (Millennium RNA markers) were purchased from Thermo Fisher Scientific Co. Expression of HCV proteins was performed *via* the transfection of HCV-G4 KM short or HCV-G4 KM long plasmids with recombinant vaccinia virus carrying T7 RNA polymerase (Shuman et al., 1988) or the transfection of purified RNA via electroporation (Gene Pulser, BioRad Co.) into HuH7/K4 cells (Salem et al., 2013). HCV proteins were detected using specific antibodies (Tsukiyama-Kohara et al., 2004), namely core (MoAb #3–12), E1 (rabbit polyclonal Ab RR3), E2 (rabbit polyclonal Ab, RR6), NS2 (rabbit polyclonal Ab), NS3 (rabbit polyclonal Ab, R212), NS4A/B (rabbit polyclonal Ab, RR12), NS5A (MoAb 32-2), and NS5B (MoAb 14-5) (Tsukiyama-Kohara et al., 2004), by western blotting or immunofluorescence assays.

### Immunohistochemistry

The sectioned liver tissue of HCV-infected chimeric mice in OCT blocks was fixed with cold acetone at −20°C for 5 min, washed once with PBS, treated with 0.03% H_2_O_2_ for 10 min at room temperature, blocked with Odyssey blocking buffer (LI-COR Co.), and reacted with anti-HCV core RR8 (Ishida et al., 2001) biotinylated antibody and streptavidin-Alexa488. Stained tissues were observed using fluorescent microscope (BZ-X700, Keyence Co.).

### Construction of Hepatitis C Virus -G4 Expressing NL Reporter and Replication Assay

To monitor replication, the HCV-G4 NL reporter clone was constructed by digesting HCV-G4KM long or G4 KM short clones with *Nsi*I and inserted in the NL gene fused at its C-terminus to the foot and mouth disease virus (FMDV) 2A autoprotease after amplification with primers (IF-G4p7-nLuc-F21 5′-gcccgaaagagcttatgcaATGAACTCCTTCTCCACAAGC-3′, IF-G4NS2-2A-R20 5′-cacctcctgatcataGGGCCCTGGGTTGGACT CGA-3′) using PCR targeting a region between p7 and NS2, as described (Shimakami et al., 2011; Yamane et al., 2014), with the In Fusion-HD cloning kit. Transfection of HCV-G4 NL reporter RNA was performed by electroporating 10 μg of *in vitro* transcribed viral RNA into 5 × 10^6^ Huh-7.5 cells stably expressing SEC14L2 (Saeed et al., 2015) with a Gene Pulser Xcell Total System (250V, 950 μF and 50 Ω) (Yamane et al., 2014). Secreted NL activity was measured in 20 μL aliquots of the supernatant fluids using the Nano-Glo Luciferase Assay System (Promega) according to the manufacturer’s protocol. The luminescent signal was measured using a Mithras LB940 Multimode Microplate Reader (Berthold).

### Ethical Statement

All experimental protocols in this study, including animal experiments, were approved by the regional ethics committee of Kagoshima University (K28002), Tokyo Metropolitan Institute of Medical Science, and Phoenix Bio Co. Patient samples were obtained according to the Declaration of Helsinki and approved by the Suez Canal University (Egypt).

## Results

### Infection of Chimeric Mice Harboring Humanized Livers With Patient Sera

**FIGURE 2:**
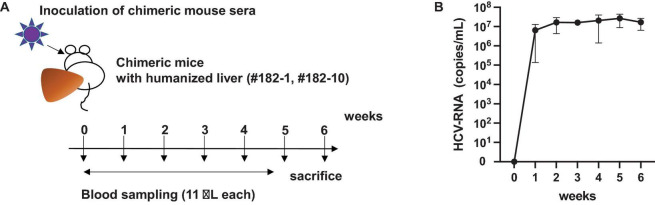
Infection of chimeric mouse. (A) Which was amplified once in chimeric mouse with a humanized liver. The chimeric mouse was bled every week and sacrificed after 6 weeks of infection. (B) HCV RNA amount in two chimeric mouse serum samples (PXB182-1, PXB182-10) was quantitated by qRT-PCR, and their average values were indicated. Vertical bars indicate the standard deviation (SD).

SCID mice transplanted with normal human hepatocytes carrying a plasminogen activator transgene (*Alb-uPA*) are highly susceptible to HCV infection (Mercer et al., 2001). Chronic hepatitis C patient plasma (10^4^ copies, genotype 4a) (Katsume et al., 2013) was intravenously injected into chimeric mice with humanized livers and used as infection source for naive chimeric mice (Figure 2). HCV-infected chimeric mice were bled weekly to obtain sera (Figure 2A) and measured for HCV RNA quantities. HCV RNA levels reached 1.7 × 10^7^ copies/mL after 6 weeks (#182) (Figure 2B), and this HCV propagation level was higher than that obtained with genotype 1a, 1b, 3a, or 6a (10^4–6^ copies/mL after 6 weeks) (Mercer et al., 2001).

### Construction of a Full Genome Hepatitis C Virus -Genotype 4a Clone

**FIGURE 3:**
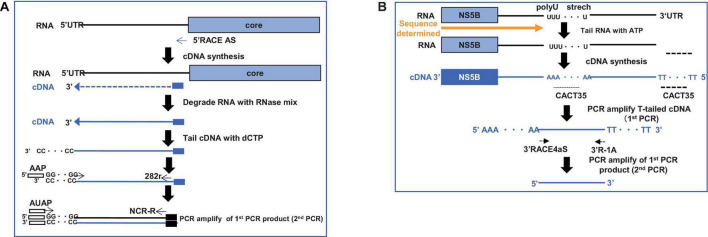
Strategy to clone HCV-G4 5′ and 3′ terminal regions. (A) Schematic of 5′ RACE strategy to clone 5′-terminal region of HCV-G4. (B) Schematic of 3′ RACE strategy to clone 3′-terminal region of HCV-G4.

After obtaining sufficient quantities of mouse sera containing the infectious HCV genotype 4a, we started the cloning process. To obtain the entire 5′UTR region, we performed 5′RACE and obtained 30 clones (Figure 3A). Three clones contained the full-length sequence of the 5′UTR, which was 341 bases. Next, we obtained the 3′UTR using the 3′RACE method and obtained a 177-nucleotide sequence (Figure 3B).

We amplified the HCV genome with high-fidelity DNA polymerase; the amplification region was divided into four sections (fragments A, B, C, D and 3′X; Figure 1A) and sub-cloned into the pSMART vector. As a result of cloning of the fragment D, we obtained a 71-nucleotide longer stretch of poly(U) sequence in the 3′UTR in Fragment G than that of the HCV-G4 KM short clone (Figure 1B). We determined the entire sequence of this clone and named it HCV-G4 KM short (9,545 nucleotides, GenBank: AB795432.1, Figure 1A and Supplementary Figure 1). We also obtained a 71-nucleotide longer 3′-UTR sequence than that of the HCV-G4 KM short clone, and the 3′UTR of this clone was 249 nucleotides (HCV-G4 KM long; 9,617 nucleotides; GenBank: AB795432.2, Figure 1B and Supplementary Figure 2).

### Construction of the Infectious HCV-Genotype 4a Clone

**FIGURE 4:**
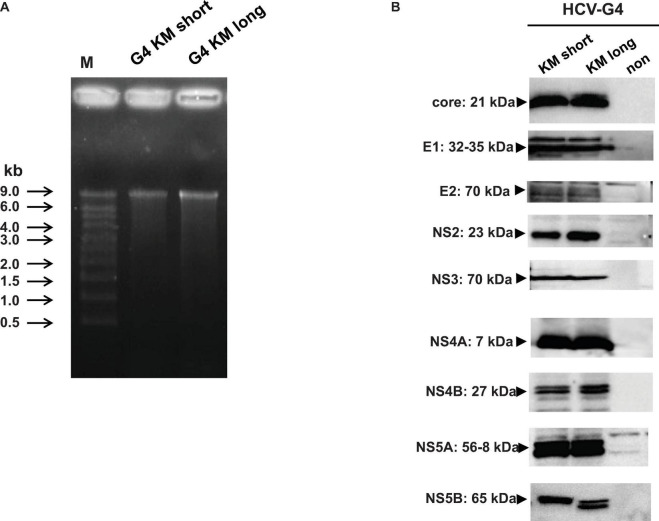
Expression of HCV-G4 KM short or HCV-G4 KM long clone *in vitro*. (A) Transcripts of HCV-G4 KM short or HCV-G4 KM long (1 μg/lane) were examined in a formamide agarose gel. (B) Detection of HCV proteins in HCV-G4 KM short or HCV-G4 KM long-expressing cells, which was induced by infection with T7 RNA polymerase encoding vaccinia virus. Representative results of three experiments are shown.

Next we synthesized the RNA from HCV-G4 KM short and HCV-G4 KM long clones using T7 RNA polymerase and examined their lengths *via* formaldehyde agarose gel electrophoresis (Figure 4A). The expression of viral proteins from the HCV-G4 KM short and HCV-G4 KM long clones was determined by T7 vaccinia virus infection and examined by immunoblotting using specific antibodies. As a result, all HCV proteins were expressed with an appropriate size (Figure 4B).

**FIGURE 5:**
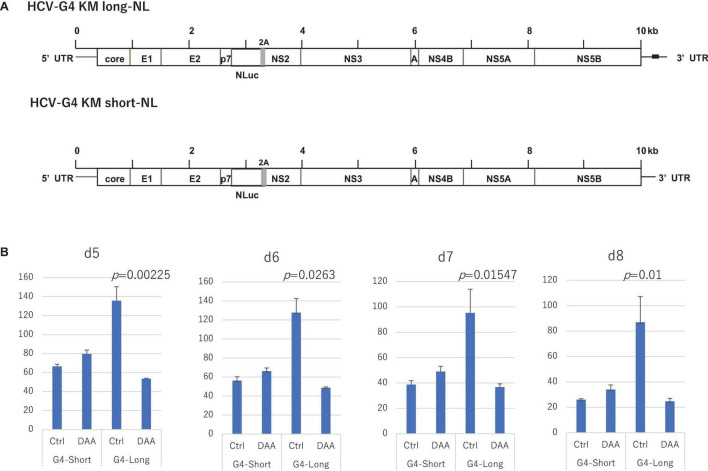
Construction of NL-containing HCV-G4 expression vectors, HCV-G4 KM long-NL and HCV-G4 KM short-NL (A). Poly (U) stretch in 3′UTR of HCV-G4 KM long is indicated as a black box. (B) These clones were used to infect Huh-7.5 cells expressing SEC14L2 and measured after day 5, 6, 7 and 8 (*n* = 4). Their replication activity was measured by NL assays. Treatment with DAA (30 μM sofosbuvir) decreased their replication. Significant differences based on the control were evaluated by statistical analysis (*t*-test, 2-sided), and a *p*-value less than 0.05 was considered significant.

To further define the replication activity of HCV-G4 KM clones, we constructed NL-inserted chimeric clones (Figure 5A). After 5–8 days of transfection into Huh-7.5 cells expressing SEC14L2 (Yamane et al., 2014), we observed replication of the G4 KM long clone, which could be significantly suppressed by the treatment with DAA. In contrast, the G4 KM short clone failed to replicate to detectable levels (Figure 5B).

**FIGURE 6:**
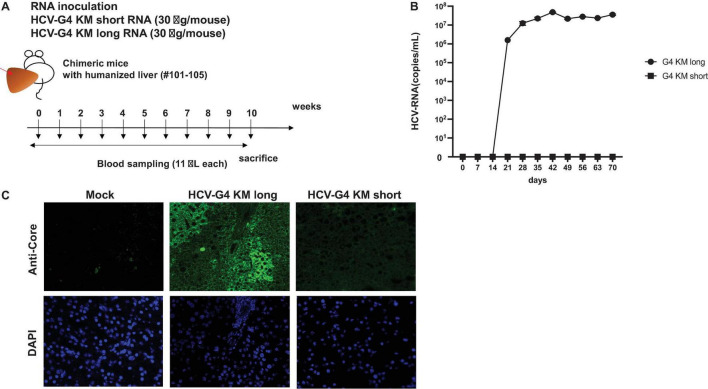
HCV-G4 KM short or HCV-G4 KM long infection in humanized chimeric mouse. (A) Purified HCV-G4 KM short or HCV-G4 KM long RNA (30 μg/mouse) was intrahepatically injected into humanized livers of chimeric mice (male, 20-weeks-old), which were bled every week until 10 weeks. (B) HCV RNA average amounts (copies/mL) in chimeric mouse sera (*n* = 2) were monitored in triplicate samples by qRT-PCR. Vertical bars indicate the SD. (C) Staining of mock-infected (PXB288-032), HCV-G4KM long-infected (PXB201) or HCV-G4 KM short infected (G4KMN1-101) chimeric mouse liver tissue (×200) with anti-core antibody (RR8). Representative data of triplicate samples are shown.

HCV-G4 KM short or HCV-G4 KM long RNA was synthesized *in vitro*, and purified RNA was intra-hepatically injected into chimeric mice (30 μg/mouse; Figure 6A). Mice were bled every week for 10 weeks and then sacrificed. HCV RNA could not be detected in any of the sera obtained from chimeric mice injected with HCV-G4 KM short RNA.

After injection of the HCV-G4 KM long RNA, the level of HCV RNA in chimeric mouse sera showed a significant increase after 3 weeks post-infection (wpi; *t*-test, *p* < 0.05, 1.59 × 10^6^ copies/mL), reaching 4.84 × 10^7^ copies/mL at 6 wpi and it was continuously detected until 10 wpi (3.57 × 10^7^ copies/mL, Figure 6B). HCV RNA was isolated from chimeric mouse sera and quantitated by RT-PCR at 6 wpi. We also confirmed the same sequence as that of HCV-G4 KM long by sequencing the isolated RNA. HCV core protein was only detected in HCV-G4 KM long infected chimeric mouse liver tissue (Figure 6C). These results indicated infectivity in naïve chimeric mice. Together, the results of this study show the replication activity of the HCV-G4 KM long sequence *in vivo*.

## Discussion

The results of this study show the significance of the 3′UTR region in HCV-G4 infection. The HCV-G4 KM long clone has a 3′UTR that is 71-nucleotides longer than that of the HCV-G4 KM short clone. The HCV-G4 KM long clone can replicate *in vitro* and *in vivo*, but the HCV-G4 KM short clone could not. Thus, this study highlighted the importance of the 3′UTR for HCV genotype 4a viral infectivity.

The reason why HCV-G4 KM short was abundantly cloned or its characteristics as a viral clone are still unclear at present. It might be due to the higher cloning efficiency of HCV-G4 KM short than HCV-G4 KM long. A previous study reported that deletion of the poly(U/UC) region in the 3′UTR decreases the suppressive effect of NS5A on translation of the full-length HCV genomic RNA (Hoffman et al., 2015). This suggests that translation of HCV-G4 KM short could be more pronounced than HCV-G4 KM long. When compared nucleotide sequence of G4-KM short and long, there were two silent mutations (No. 8939 G to A, No. 9080 G to A (Supplementary Figure 3A) in the coding region, which did not cause amino acid changes. In addition, the 3′UTR ply(U/C) stretch is 71 nucleotides shorter in HCV-G4 KM short than the long clone (Supplementary Figure 3B).

The entire nucleotide homology of HCV-G4 KM long with that of an *in vitro* and *in vivo* infectious HCV JFH-1 clone (9,678 nucleotide, GenBank AB047639.1) was 62% (Wakita et al., 2005). The 5′UTR of the JFH-1 clone was 341 nucleotides, and the 3′UTR was 236 nucleotides (Table 2). The 5′UTR of HCV-G4 KM long was 341 nucleotides, and the length of the 3′UTR was 249 nucleotides.

**TABLE 2:** Comparison of 3′UTR of G4-KM long with that of other HCV infectious clones.

Clone	Nucleotide position	Length (nt)	Poly(U)
G4-KM long	9,369–9,617	249	9,400–9,481
ED43	9,368–9,579	212	9,401–9,446
JFH-1	9,414–9,650	236	9,482–9,539

The HCV-G4 KM long clone showed 80–90% nucleotide homology with previously reported HCV genotype 4 clones. For example, *in vivo* infectious HCV genotype 4a clone, HCV ED43 strain (Genbank GU814266.1, 9,579 nucleotides), shows 91% homology with that of HCV-G4 KM long (Gottwein et al., 2010). The length of the 5′UTR is 340 nucleotides and that of 3′UTR is 212 nucleotides in HCV ED43 (Table 2). The HCV ED43 strain does not show infectivity in HuH-7 cells; however, it did shows infectivity in chimpanzees (Gottwein et al., 2010). The HCV-G4 KM long nucleotide sequence showed 85% homology with the QC352 strain (9,431 nucleotides) (Lu et al., 2015), 81% homology with QC147 (9,426 nucleotides), 80% homology with QC361 (9,426 nucleotides), 80% homology with HCV subtype 4f strain IFBT88 (9,304 nucleotides) or IFBT84 (Hmaied et al., 2007), and 80% homology with the CYHCV048 strain (9,174 nucleotides) (Demetriou and Kostrikis, 2011). However, none of these clones contained a longer 3′UTR sequence than that of the HCV-G4 KM long clone.

## Conclusion

The HCV-G4 KM long clone is an infectious clone *in vitro* and *in vivo*. The infectious HCV genotype 4a clone and chimeric clones with NL established in this study should help to characterize the biological features and clarify the molecular basis of the pathogenesis of HCV genotype 4a.

## Data Availability Statement

The original contributions presented in the study are included in the article/Supplementary Material, further inquiries can be directed to the corresponding author/s.

## Ethics Statement

The studies involving human participants were reviewed and approved by the Egypt-Japan University for Science and Technology. The patients/participants provided their written informed consent to participate in this study. The animal study was reviewed and approved by the Kagoshima University and Tokyo Metropolitan Institute.

## Author Contributions

KT-K and MK designed the experiments, reviewed the data, planned the experimental strategy, and wrote the manuscript. AT, DY, BK, YT, and YA performed the experiments. AE-G obtained patient samples and helped in the experimental design. All authors contributed to the article and approved the submitted version.

## Conflict of Interest

The authors declare that the research was conducted in the absence of any commercial or financial relationships that could be construed as a potential conflict of interest.

## Publisher’s Note

All claims expressed in this article are solely those of the authors and do not necessarily represent those of their affiliated organizations, or those of the publisher, the editors and the reviewers. Any product that may be evaluated in this article, or claim that may be made by its manufacturer, is not guaranteed or endorsed by the publisher.

## Supplementary Material

Supplementary Figure 1 Nucleotide sequence of HCV-G4 KM short clone GenBank: AB795432.1.

Supplementary Figure 2 Nucleotide sequence of HCV-G4 KM long clone GenBank: AB795432.2.

Supplementary Figure 3 **(A)** Comparison of HCV-G4 KM long and short nucleotide sequences and substituted nucleotides within coding region were shown. Nucleotide substitutions in coding region without amino acid changes are indicated by the red color. Other sequences were all conserved between HCV-G4 KM long and short. **(B)** Comparison of HCV-G4 KM long and short and nucleotide differences within the 3′UTR were shown. The translation stop codon is indicated with a red square, and positions of U-stretch and 3′X regions are indicated.
