# Supplementary material for: Longer Poly(U) Stretches in the 3′UTR Are Essential for Replication of the Hepatitis C Virus Genotype 4a Clone in in vitro and in vivo
Source: Front Microbiol. 2021 Nov 25;12:764816. doi: 10.3389/fmicb.2021.764816 (PMC8656456; doi:10.3389/fmicb.2021.764816)
Supplement: Supplementary Figure 1 — Nucleotide sequence of HCV-G4 KM short clone GenBank: AB795432.1. [file Table_1.pdf]

Fig.S1

Nucleotide sequence of HCV-G4 KM short clone GenBank: AB795432.1

Gene 1…9545

5'UTR 1…341

CDS 342…9368

1 acctgctctc tatgagagca acactccacc atgaaccgct cccctgtgag gaactactgt

61 cttcacgcag aaagcgtcta gccatggcgt tagtatgagt gttgtacagc ctccaggatc

121 ccccctcccg ggagagccat agtggtctgc ggaaccggtg agtacaccgg aatcgccggg

181 atgaccgggt cctttcttgg attaaacccg ctcaatgccc ggaaatttgg gcgtgccccc

241 gcgagactgc tagccgagta gtgttgggtc gcgaaaggcc ttgtggtact gcctgatagg

301 gtgcttgcga gtgccccggg aggtctcgta gaccgtgcac catgagcacg aatcctaaac

361 ctcaaagaaa aaccaaacgt aacaccaacc gccgccccat ggacgtcaag ttcccgggtg

421 gtggccagat cgttggcgga gtttacttgt tgccgcgcag ggcccccga ttgggtgtgc

481 gcgcgactcg gaagacttcg gagcggtcgc aacctcgtgg gagacgccaa cctatcccca

541 aggcgcgtcg atccgaggga aggtcctggg cacagccagg atatccatgg cctctttacg

601 gtaatgaggg ttgcgggtgg gcaggatggc tcttgtcccc ccgtggctct cgaccgtctt

661 ggggcccaaa tgatccccgg cggaggtccc gcaatttggg taaggtcatc gataccctaa

721 cctgcggctt cgccgacctc atgggataca tcccggtcat aggcgccccc gtgggtggcg

781 tcgccagggc cctagcacat ggtgtcaggg ccgtggagga cgggatcaat tacgcaacag

841 gaaatcttcc cggttgctcc ttttctatct tcctcttggc acttctctcg tgcctgactg

901 tccccgcttc ggccatcaac tatcgcaacg tttcgggtat ttaccacgtc accaatgact

961 gcccgaactc aagcatagtg tatgaggccg accatcacat cttgcatctt ccaggttgcg

1021 tgccctgcgt gaagacgggg aatcagtcac gttgctgggt ggcccttact cctaccgtcg

1081 cagcgccgta catcggtgcg ccacttgagt ctctacggag tcatgtggat ttgatggtgg

1141 gggctgccac tgcttgctca gccctttaca tcggggactt gtgtggcggc ttgttcctgg

1201 tcggtcagat gtttactttc cgaccacggc gccactggac cacccaggaa tgcaattgtt

1261 ccatctatac agggcacatc actggccaca gaatggcctg ggacatgatg atgaactgga

1321 gtccaacaac caccttaatt ctcgcccagg tcatgaggat cccggggact ctgctagact

1381 tgcttgcagg gggccactgg ggtgtcctcg tgggaatagc ctactacagc atgcaggcta

1441 attgggctaa agtcatcttg gtcctattcc tttttgcagg ggttgacgcc gagactcatg

1501 tgactggggg ggtagtcggt cgcagtgtct ccagctttac tgggctcttt aggcttggat

1561 cccagcagaa tgtgcagctc atcaacagca acgggagctg gcatataaat aggacagccc

1621 tcaattgcaa tgatagccta aatactgggt tcttggctgc cttgttctac accaacaggt

1681 ttaacagctc agggtgtact gaacggctcg caagctgcaa gagccttgac agctatgacc

1741 aaggctgggg tccgctcggg gtcgccaaca tcagcggccc gtccgatgac aaaccctact

1801 gctggcacta cgcgcctcgg tcgtgcggga tcgtgccagc gtccagtgtg tgtggtcccg

1861 tgtattgctt cactcccagc cctgtcgtgg tcggaaccac cgatcgcttc ggggtcccta

1921 cttacacctg gggggagaat gagtctgatg tcttcctttt gaactcgacc agaccgccgc

1981 atggtgcgtg gtttggatgt gtgtggatga acggtaccgg gtttacaaaa acctgtggcg

2041 ctcctccatg caaagtcaac accagcaata acacctggca ctgccccact gactgtttca

2101 ggaagcatcc tgagactacc tatgccaagt gcgggtcagg tccttggatc acgccacgat

2161 gcttgatcga ttacccgtac cggctgtggc attacccatg caccgtcaac ttcaccatct

2221 ttaaggtcag aacctttgtc ggcggtatag agcatcggtt gcaagcagca tgcaactgga

2281 ccaggggggga agcctgcggc ttggagcata gagatcgcgc agaactgtcg cctctgctcc

2341 ttaccactac aacgtggcag gtcctcccct gctctttcac cactctacct gccctttcta

2401 ccggcctgat ccacctccac caaaacatcg tggacgttca atacctctac ggtgttggat

2461 ccgcggtggt gtcttgggcc cttaaatggg aatatgtggt actcgcgttc ctgcttctcg

2521 cagacgcaag agtctctgct tgcctatgga tgatggttat ggtagctcag gttgaggcgg

2581 ctttgtccaa cctaatcaac atcaacgctg cttcagccgc tggcactcaa agcttctggt

2641 atgccattct cttcatctgc attgcttggc atgtcaaggg ccggctcccg gctattgccg

2701 cttacgcggc ctgcgggatg tggcccctgc ttctcctgct tctaatgctg cccgaaagag

2761 cttatgcata tgatcaggag gtggcagggt cccttggcgg cgctgtcgtt gtcatgctga

2821 ccatcctgac attgtctcca cactacaagt catggctggc tcggggattg tggtggattc

2881 aatatttcat agctaggacc gaggccacgc tgcatgtcta tgttccatcc ttcgacgtgc

2941 gcggacctcg cgactcactg attattcttg cggtcctggc ctgtccacat ttggtttttg

3001 acatcacaaa atatctcctg gccatcatag ggcccctcta tgtactccag gcctcactcc

3061 tacgcgtccc ttactttgtg agggcacacg cgctggttaa gatctgtggc ttgttgcgag

3121 gggtggtctg tggcaaatat tgccaaatgg ccgtgcttaa agtgggggcc ttgactggta

3181 cttacatcta tgaccacctt actcccctgt cagactgggc cgctgagggt cttcgagact

3241 tggcggtggc cctggagccg gttgtgttca cgcccatgga gaagaaaatc atcgtctggg

3301 gcgctgacac cgctgcgtgt ggcgacatca taggggggcct gcctgtctcg gctaggttgg

3361 gcaatgagat cttgctcggg cctgccgact cagaaacatc aaaggggtgg agactccttg

3421 cccccatcac agcgtacgcg cagcagaccc gcggcttgtt cagtaccatc gtaacgagcc

3481 tcactggcag ggacaccaat gagaactgtg gtgaagtcca ggtcctatct accgccacgc

3541 aatccttcct gggtacagcg gttaacggcg tgatgtggac cgtctaccac ggggcgggta

3601 gcaaaaccat tagcggccca aagggacctg tcaaccaaat gtacaccaat gttgaccaag

3661 acttggtggg gtggccagcg ccccccggag tcagatctct tacgccatgc acctgcggtg

3721 cgtcggactt gtacctagtc actaggcacg ccgatgtggt gcccgtgcgc aggagaggag

3781 acactagagg agctctcttg agccctagac caatatccac tcttaagggg tcttctggtg

3841 gtccgctgct ctgccccatg ggacacgccg ccggcatatt tcgcgcggcg gtgtgtactc

3901 gaggggtggc caaggcagtg gactttgttc cagttgagtc ccttgagacc actatgagat

3961 caccagtgtt cactgacaat tcaacacccc cagcggtgcc tcagacctat caggtcgcgc

4021 acctgcacgc accaacaggg agtggtaaga gcaccaaggt cccggcagcg tacgctgccc

4081 agggctataa ggtgctagtg ctcaacccct cagttgcggc cacactgggg tttggggtat

4141 acatgtccaa ggcatatggt attgacccga acattcggtc aggagtcagg accatcacca

4201 cgggtgcgcc aatcacgtac tctacatacg gtaagttcct ggccgatgga ggttgcagcg

4261 gggggcata tgacataatc atctgtgacg agtgccactc caccgactcc acaacaatcc

4321 ttggcatagg cacggtcctg gaccaagcgg agaccgctgg agcacggctc gtcgtgctcg

4381 cgaccgctac ccccgccaggg tcggtgacca caccccattc caacatagag gaggtcgccc

4441 tgcctacgac gggagaaata cccttctatg gcagggcgat ccccctagag ctaattaagg

4501 ggggcagaca tctcatcttc tgtcactcga agaaaaaatg tgatgaactg gctaaacaac

4561 tgacatctct aggtctaaat gctgtagcct actacagagg cttagacgtt tcggtgatcc

4621 ctgcgtctgg ggacgtcgtg gtatgtgcca cggacgccct catgacaggt tttaccggcg

4681 actttgactc agtgatagac tgcaatacat ctgtgataca gactgttgac ttcagcttgg

4741 accccacctt ctccatagag actacaactg ttcctcagga cgcggtatcc cgcagtcagc

4801 ggagaggccg cactggtagg ggaaggttag gcacataccg gtatgtcacc ccaggggaga

4861 gaccatcagg catatttgac acctcagtgc tctgcgagtg ctacgacgcc ggatgcgcct

4921 ggtacgagct gacaccggct gagaccacaa caaggctgag agcttacttc aacacaccag

4981 gccttcctgt gtgccaagac cacctggagt tctgggagag cgtctttaca gggctaaccc

5041 aaatagacgg tcatttccta tcccagacca aacaagcggg cgataacttc ccataccttg

5101 tcgcttatca agcgacggtg tgtgccagag ctatggcgcc ccctccaagt tgggacacca

5161 tgtggaagtg cctaatccgc ctcaagccta ccctgcacgg gcctacgccc cttctctata

5221 gattggggtc tgtgcagaat gaggtgacac tcacccatcc catcactaaa tacatcatgg

5281 cttgcatgtc agctgacctt gaggtagtga cgagcacgtg ggttctggtg ggcggcgtcc

5341 tggcagctct ggctgcttac tgtctctcag tgggcagcgt agtgattgtc gggagagtcg

5401 tcctgtcagg ccaacctgct gtcattcctg accgcgaagt gctctaccaa cagttcgacg

5461 aaatggaaga gtgttccaaa caccttccac tagtcgagca tgggttacaa ctggctgagc

5521 agttcaagca gaaggccgta ggccttctaa acttcgctgg caagcaagcc caagaggcaa

5581 caccagtaat ccagtccaac ttcgccaaac tcgaacagtt ctgggcgaag cacatgtgga

5641 atttcatcag cggcattcaa tatctcgctg gcctgtccac cttgcctggc aatccggcta
5701 ttgcttccct catgtccttc actgccgccg ttacaagccc cctgaccact cagcaaaccc
5761 tcctttttaa catcttaggg ggatgggtgg cctcgcagat cgcgactccg acagcttcta
5821 ccgcatttgt cgtgagcggt cttgcagggg cggcagttgg cagtgtgggc cttggcaaga
5881 ttctggtgga cattctcgcc ggttacggcg ccggcgtagc tggcgccgtg gtcaccttca
5941 aaatcatgag cggcgagatg ccatccacag aggacttggt gaacctgctc ccggccattc
6001 tatcgcctgg agcactggta gtaggggtgg tatgtgcggc aatcttgcgc cgccacgtgg
6061 gcccaggtga aggggccgtg cagtggatga accgtctaat tgcgttcgca tcgcgaggca
6121 atcacgtgtc tcccacgcac tacgtccctg agtctgacgc agcagctcgc gtgacccaga
6181 tactatcatc cctcactgtg acatctcttc tcagacgcct ccacaagtgg atcaatgaag
6241 attgctccac tccatgtgcc gaatcttggc tatgggaggt gtgggattgg gtctgcactg
6301 tgctgagtga cttcaagacg tggctaaaag ccaagctgct acccctcatg ccaggtattc
6361 ccttcctctc gtgccagagg ggctataggg gagagtggcg tggggatggc gtgatgcaca
6421 ccaaatgccc ctgcggagca gagttggcag gccacatcaa gaacggctcg atgagaatca
6481 ctgggccgaa aacttgcagc aacacatggc atggtacctt ccccattaat gcttacacca
6541 caggccccgg cgtgcccatc ccagcgccga actacaagtt cgcgctttgg agggtgtccg
6601 ctgaggagta cgtggaggtt cgcagagtgg gtgatttcca ttatgtcacc ggggtaacac
6661 aagacaacat caagtgcccc tgccaagttc cggctccaga gttcttcaca gaggtggacg
6721 gcatcaggct acaccgccac gccccgaagt gcaaaccctt gctgcgggaa gaagtgtcgt
6781 tctcagtggg actcaattcg ttcgtggtgg gatcacaact cccatgcgag ccagagccgg
6841 acgtggccgt gctaacatcc atgctgacag acccatccca cataacggcg gaagcggcga
6901 gccggagact agctcgaggg tcaccgccct cattagctag ttcctcggcg agccagctgt
6961 cccgcccttc tctcaaggcc acatgcaccg ctcatcatga ctcccctggt gttgatctcc
7021 tcgaggctaa cctcttgtgg gggtctaccg ctaccagggt cgagacaaat gagaaggtga
7081 taatactgga ctcttttgag ccatgtgtgg ctgaaccaga tgatgacagg gaggtctcgg
7141 ttgccgcaga aatcctgcgt ccgaccaaga agttccctcc agctctacca atctgggctc
7201 gaccagatta caatccacct ctcactgaga cgtggaagca acaggactac gagcctccga
7261 ccgtccacgg gtgtgctctg ccccccagca agcaaacccc cgttcctcct cccaggagga
7321 agaggacggt tcagctcact gagtccgttg ttaccactgc tttggcagag ctggccgcga
7381 agaccttcgg ccagtcagag ctgggctcgg actccggcgc agacctcgcc accccaactg
7441 agaccacaga ctcgggcccc ctcattgtgg atgacgcatc cgatgacgga tcttattcgt
7501 caatgcctcc actagagggg gagcccggtg acccggactt ggcatcagat tcttggtcca
7561 ctgttagcgg atcagaggac gtcgtgtgct gctcaatgtc gtactcatgg actggggcgc

7621 ttgtgacacc ttgcgcggct gaggaatcaa agctgccaat tagccccctg agcaattcac

7681 tcttacgcca tcacaacatg gtgtatgcca cgaccacccg ttctgccgta acccggcaga

7741 agaaggtgac ctttgaccgc ctgcaggtgg tagacaacca ttacaatgat gtactcaagg

7801 agatcaaagc acgagcatcc agagtgaagg cacgcttgct taccacggag gaagcttgcg

7861 acctgacgcc cccccactct gccaaatcga aattcggcta tggggcgaag gatgttcgta

7921 gctattcccg caaggccatt aaccacatca actccgtgtg ggaggacttg ctggatgaca

7981 acaatactcc aataccaaca acaatcatgg ccaaaaatga ggttttcgtt gtgaacgcag

8041 cgaagggagg tcggaagcct gcccgcctga tcgtgtatcc ggatcttggg gttcgggttt

8101 gcgagaagag ggcgcttcac gacgtcgtca aacagctacc tgaggccgtg atgggagccg

8161 cttacggctt ccagtactcc ccagcgcagc gggtagattt tcttctgact gcttggaagt

8221 caaagaagaa tcctatgggg ttctcttatg acacccgctg ctttgactcc actgtaaccg

8281 aaaaggacat cagagccgag gaagaggtct atcagtgttg tgacctggag cccgaagccc

8341 gcaaggttat tgccgccctc acagagagac tctacgtggg cggccccatg tataacagca

8401 agggagacct gtgtgggtat cggagatgcc gtgcaagcgg cgtctacacc accagcttcg

8461 gaaacacact gacgtgctat ctcaaagcta cagccgctat tagggcggcc gggctgagag

8521 actgcactat gctggtttgc ggcgacgact tagtcgtcat cgctgagagc gacggtgttg

8581 aggaggataa ccgaggtctc cgagccttcg cagaggctat gacgagatac tcggctcccc

8641 caggcgacgc cccgcaacca gcatatgacc tggaactaat aacatcatgt tcctccaacg

8701 tctcagtcgc gcacgacgcg acgggtaaga gggtatacta cctgacccga gaccctgaga

8761 cacccttggc gcgagccgca tgggagacag tccgacacac tccagtcaat tcctggttgg

8821 gaaacatcat agtctacgcc cccacaatat gggtgcgcat ggtattgatg acccactttt

8881 tctcaatact ccaaagccag gaagcccttg agaaagcact cgacttcgat atgtacgggg

8941 tcacttactc tatcactccg ctggatctac cggcaatcat tcaaagactc catggcttga

9001 gcgcatttac actgcacgga tactctccac acgaactcaa tcgggtgtct ggaagcctca

9061 ggaaacttgg ggtacccccg ttgagagcgt ggagacatcg ggcccgagca gtccgcgcca

9121 agcttattgc ccagggaggt aaggccaaaa tctgtggcat atacctcttt aactgggcag

9181 taaaaaccaa gcttaaactc actccattgc ctgccgctgc caaactcgat ttatcgggtt

9241 ggttcacggt gggcgccggc gggggagaca tttatcacag catgtctcat gcccgacccc

9301 gctatttact cctgtgccta ctcctacttt cagtaggggt aggcattttc ctgctgcctg

9361 ctcggtaggc agcttaacac tccgacctta gggtccctgt tttttttttt ctttttcctt

9421 cttttctctt cttaatcctt ctttcttggt ggctccatct tagccctagt cacggctagc

9481 tgtgaaaggt ccgtgagccg catgactgca gagagtgctg aaactggcct ctctgcagat

9541 catgt
