# Supplementary material for: Longer Poly(U) Stretches in the 3′UTR Are Essential for Replication of the Hepatitis C Virus Genotype 4a Clone in in vitro and in vivo
Source: Front Microbiol. 2021 Nov 25;12:764816. doi: 10.3389/fmicb.2021.764816 (PMC8656456; doi:10.3389/fmicb.2021.764816)
Supplement: Supplementary Figure 2 — Nucleotide sequence of HCV-G4 KM long clone GenBank: AB795432.2. [file Table_2.pdf]

Fig.S2

Nucleotide sequence of HCV-G4 KM long clone GenBank: AB795432.2

Gene 1…9617

5'UTR 1…341

CDS 342…9368

```
   1 acctgctctc tatgagagca acactccacc atgaaccgct cccctgtgag gaactactgt
  61 cttcacgcag aaagcgtcta gccatggcgt tagtatgagt gttgtacagc ctccaggatc
 121 ccccctcccg ggagagccat agtggtctgc ggaaccggtg agtacaccgg aatcgccggg
 181 atgaccgggt cctttcttgg attaaacccg ctcaatgccc ggaaatttgg gcgtgccccc
 241 gcgagactgc tagccgagta gtgttgggtc gcgaaaggcc ttgtggtact gcctgatagg
 301 gtgcttgcga gtgccccggg aggtctcgta gaccgtgcac catgagcacg aatcctaaac
 361 ctcaaagaaa aaccaaacgt aacaccaacc gccgccccat ggacgtcaag ttcccgggtg
 421 gtggccagat cgttggcgga gtttacttgt tgccgcgcag ggcccccgga ttgggtgtgc
 481 gcgcgactcg gaagacttcg gagcggtcgc aacctcgtgg gagacgccaa cctatcccca
 541 aggcgcgtcg atccgaggga aggtcctggg cacagccagg atatccatgg cctctttacg
 601 gtaatgaggg ttgcgggtgg gcaggatggc tcttgtcccc ccgtggctct cgaccgtctt
 661 ggggcccaaa tgatccccgg cggaggtccc gcaatttggg taaggtcatc gataccctaa
 721 cctgcggctt cgccgacctc atgggataca tcccggtcat aggcgccccc gtgggtggcg
 781 tcgccagggc cctagcacat ggtgtcaggg ccgtggagga cgggatcaat tacgcaacag
 841 gaaatcttcc cggttgctcc ttttctatct tcctcttggc acttctctcg tgcctgactg
 901 tccccgcttc ggccatcaac tatcgcaacg tttcgggtat ttaccacgtc accaatgact
 961 gcccgaactc aagcatagtg tatgaggccg accatcacat cttgcatctt ccaggttgcg
1021 tgccctgcgt gaagacgggg aatcagtcac gttgctgggt ggcccttact cctaccgtcg
1081 cagcgccgta catcggtgcg ccacttgagt ctctacggag tcatgtggat ttgatggtgg
1141 gggctgccac tgcttgctca gccctttaca tcggggactt gtgtggcggc ttgttcctgg
1201 tcggtcagat gtttactttc cgaccacggc gccactggac cacccaggaa tgcaattgtt
1261 ccatctatac agggcacatc actggccaca gaatggcctg ggacatgatg atgaactgga
1321 gtccaacaac caccttaatt ctcgcccagg tcatgaggat cccggggact ctgctagact
1381 tgcttgcagg gggccactgg ggtgtcctcg tgggaatagc ctactacagc atgcaggcta
1441 attgggctaa agtcatcttg gtcctattcc tttttgcagg ggttgacgcc gagactcatg
1501 tgactggggg ggtagtcggt cgcagtgtct ccagctttac tgggctcttt aggcttggat
1561 cccagcagaa tgtgcagctc atcaacagca acgggagctg gcatataaat aggacagccc
1621 tcaattgcaa tgatagccta aatactgggt tcttggctgc cttgttctac accaacaggt
```

1681 ttaacagctc agggtgtact gaacggctcg caagctgcaa gagccttgac agctatgacc

1741 aaggctgggg tccgctcggg gtcgccaaca tcagcggccc gtccgatgac aaaccctact

1801 gctggcacta cgcgcctcgg tcgtgcggga tcgtgccagc gtccagtgtg tgtggtcccg

1861 tgtattgctt cactcccagc cctgtcgtgg tcggaaccac cgatcgcttc ggggtcccta

1921 cttacacctg gggggagaat gagtctgatg tcttcctttt gaactcgacc agaccgccgc

1981 atggtgcgtg gtttggatgt gtgtggatga acggtaccgg gtttacaaaa acctgtggcg

2041 ctcctccatg caaagtcaac accagcaata acacctggca ctgccccact gactgtttca

2101 ggaagcatcc tgagactacc tatgccaagt gcgggtcagg tccttggatc acgccacgat

2161 gcttgatcga ttacccgtac cggctgtggc attacccatg caccgtcaac ttcaccatct

2221 ttaaggtcag aacctttgtc ggcggtatag agcatcggtt gcaagcagca tgcaactgga

2281 ccaggggggа agcctgcggc ttggagcata gagatcgcgc agaactgtcg cctctgctcc

2341 ttaccactac aacgtggcag gtcctcccct gctctttcac cactctacct gccctttcta

2401 ccggcctgat ccacctccac caaaacatcg tggacgttca atacctctac ggtgttggat

2461 ccgcggtggt gtcttgggcc cttaaatggg aatatgtggt actcgcgttc ctgcttctcg

2521 cagacgcaag agtctctgct tgcctatgga tgatggttat ggtagctcag gttgaggcgg

2581 ctttgtccaa cctaatcaac atcaacgctg cttcagccgc tggcactcaa agcttctggt

2641 atgccattct cttcatctgc attgcttggc atgtcaaggg ccggctcccg gctattgccg

2701 cttacgcggc ctgcgggatg tggcccctgc ttctcctgct tctaatgctg cccgaaagag

2761 cttatgcata tgatcaggag gtggcagggt cccttggcgg cgctgtcgtt gtcatgctga

2821 ccatcctgac attgtctcca cactacaagt catggctggc tcggggattg tggtggattc

2881 aatatttcat agctaggacc gaggccacgc tgcatgtcta tgttccatcc ttcgacgtgc

2941 gcggacctcg cgactcactg attattcttg cggtcctggc ctgtccacat ttggtttttg

3001 acatcacaaa atatctcctg gccatcatag ggcccctcta tgtactccag gcctcactcc

3061 tacgcgtccc ttactttgtg agggcacacg cgctggttaa gatctgtggc ttgttgcgag

3121 gggtggtctg tggcaaatat tgccaaatgg ccgtgcttaa agtgggggcc ttgactggta

3181 cttacatcta tgaccacctt actcccctgt cagactgggc cgctgagggt cttcgagact

3241 tggcggtggc cctggagccg gttgtgttca cgcccatgga gaagaaaatc atcgtctggg

3301 gcgctgacac cgctgcgtgt ggcgacatca taggggggcct gcctgtctcg gctaggttgg

3361 gcaatgagat cttgctcggg cctgccgact cagaaacatc aaaggggtgg agactccttg

3421 cccccatcac agcgtacgcg cagcagaccc gcggcttgtt cagtaccatc gtaacgagcc

3481 tcactggcag ggacaccaat gagaactgtg gtgaagtcca ggtcctatct accgccacgc

3541 aatccttcct gggtacagcg gttaacggcg tgatgtggac cgtctaccac ggggcgggta

3601 gcaaaaccat tagcggccca aagggacctg tcaaccaaat gtacaccaat gttgaccaag

3661 acttggtggg gtggccagcg ccccccggag tcagatctct tacgccatgc acctgcggtg

3721 cgtcggactt gtacctagtc actaggcacg ccgatgtggt gcccgtgcgc aggagaggag

3781 acactagagg agctctcttg agccctagac caatatccac tcttaagggg tcttctggtg

3841 gtccgctgct ctgccccatg ggacacgccg ccggcatatt tcgcgcggcg gtgtgtactc

3901 gaggggtggc caaggcagtg gactttgttc cagttgagtc ccttgagacc actatgagat

3961 caccagtgtt cactgacaat tcaacacccc cagcggtgcc tcagacctat caggtcgcgc

4021 acctgcacgc accaacaggg agtggtaaga gcaccaaggt cccggcagcg tacgctgccc

4081 agggctataa ggtgctagtg ctcaacccct cagttgcggc cacactgggg tttggggtat

4141 acatgtccaa ggcatatggt attgacccga acattcggtc aggagtcagg accatcacca

4201 cgggtgcgcc aatcacgtac tctacatacg gtaagttcct ggccgatgga ggttgcagcg

4261 gggggcata tgacataatc atctgtgacg agtgccactc caccgactcc acaacaatcc

4321 ttggcatagg cacggtcctg gaccaagcgg agaccgctgg agcacggctc gtcgtgctcg

4381 cgaccgctac ccgccaggg tcggtgacca caccccattc caacatagag gaggtcgccc

4441 tgcctacgac gggagaaata cccttctatg gcagggcgat ccccctagag ctaattaagg

4501 ggggcagaca tctcatcttc tgtcactcga agaaaaaatg tgatgaactg gctaaacaac

4561 tgacatctct aggtctaaat gctgtagcct actacagagg cttagacgtt tcggtgatcc

4621 ctgcgtctgg ggacgtcgtg gtatgtgcca cggacgccct catgacaggt tttaccggcg

4681 actttgactc agtgatagac tgcaatacat ctgtgataca gactgttgac ttcagcttgg

4741 accccacctt ctccatagag actacaactg ttcctcagga cgcggtatcc cgcagtcagc

4801 ggagaggccg cactggtagg ggaaggttag gcacataccg gtatgtcacc ccaggggaga

4861 gaccatcagg catatttgac acctcagtgc tctgcgagtg ctacgacgcc ggatgcgcct

4921 ggtacgagct gacaccggct gagaccacaa caaggctgag agcttacttc aacacaccag

4981 gccttcctgt gtgccaagac cacctggagt tctgggagag cgtctttaca gggctaaccc

5041 aaatagacgg tcatttccta tcccagacca aacaagcggg cgataacttc ccataccttg

5101 tcgcttatca agcgacggtg tgtgccagag ctatggcgcc ccctccaagt tgggacacca

5161 tgtggaagtg cctaatccgc ctcaagccta ccctgcacgg gcctacgccc cttctctata

5221 gattggggtc tgtgcagaat gaggtgacac tcacccatcc catcactaaa tacatcatgg

5281 cttgcatgtc agctgacctt gaggtagtga cgagcacgtg ggttctggtg ggcggcgtcc

5341 tggcagctct ggctgcttac tgtctctcag tgggcagcgt agtgattgtc gggagagtcg

5401 tcctgtcagg ccaacctgct gtcattcctg accgcgaagt gctctaccaa cagttcgacg

5461 aaatggaaga gtgttccaaa caccttccac tagtcgagca tgggttacaa ctggctgagc

5521 agttcaagca gaaggccgta ggccttctaa acttcgctgg caagcaagcc caagaggcaa

5581 caccagtaat ccagtccaac ttcgccaaac tcgaacagtt ctgggcgaag cacatgtgga

5641 atttcatcag cggcattcaa tatctcgctg gcctgtccac cttgcctggc aatccggcta

5701 ttgcttccct catgtccttc actgccgccg ttacaagccc cctgaccact cagcaaaccc

5761 tcctttttaa catcttaggg ggatgggtgg cctcgcagat cgcgactccg acagcttcta

5821 ccgcatttgt cgtgagcggt cttgcagggg cggcagttgg cagtgtgggc cttggcaaga

5881 ttctggtgga cattctcgcc ggttacggcg ccggcgtagc tggcgccgtg gtcaccttca

5941 aaatcatgag cggcgagatg ccatccacag aggacttggt gaacctgctc ccggccattc

6001 tatcgcctgg agcactggta gtaggggtgg tatgtgcggc aatcttgcgc cgccacgtgg

6061 gcccaggtga aggggccgtg cagtggatga accgtctaat tgcgttcgca tcgcgaggca

6121 atcacgtgtc tcccacgcac tacgtccctg agtctgacgc agcagctcgc gtgacccaga

6181 tactatcatc cctcactgtg acatctcttc tcagacgcct ccacaagtgg atcaatgaag

6241 attgctccac tccatgtgcc gaatcttggc tatgggaggt gtgggattgg gtctgcactg

6301 tgctgagtga cttcaagacg tggctaaaag ccaagctgct acccctcatg ccaggtattc

6361 ccttcctctc gtgccagagg ggctataggg gagagtggcg tggggatggc gtgatgcaca

6421 ccaaatgccc ctgcggagca gagttggcag gccacatcaa gaacggctcg atgagaatca

6481 ctgggccgaa aacttgcagc aacacatggc atggtacctt ccccattaat gcttacacca

6541 caggccccgg cgtgcccatc ccagcgccga actacaagtt cgcgctttgg agggtgtccg

6601 ctgaggagta cgtggaggtt cgcagagtgg gtgatttcca ttatgtcacc ggggtaacac

6661 aagacaacat caagtgcccc tgccaagttc cggctccaga gttcttcaca gaggtggacg

6721 gcatcaggct acaccgccac gccccgaagt gcaaaccctt gctgcgggaa gaagtgtcgt

6781 tctcagtggg actcaattcg ttcgtggtgg gatcacaact cccatgcgag ccagagccgg

6841 acgtggccgt gctaacatcc atgctgacag acccatccca cataacggcg gaagcggcga

6901 gccggagact agctcgaggg tcaccgccct cattagctag ttcctcggcg agccagctgt

6961 ccgccccttc tctcaaggcc acatgcaccg ctcatcatga ctcccctggt gttgatctcc

7021 tcgaggctaa cctcttgtgg gggtctaccg ctaccagggt cgagacaaat gagaaggtga

7081 taatactgga ctcttttgag ccatgtgtgg ctgaaccaga tgatgacagg gaggtctcgg

7141 ttgccgcaga aatcctgcgt ccgaccaaga agttccctcc agctctacca atctgggctc

7201 gaccagatta caatccacct ctcactgaga cgtggaagca acaggactac gagcctccga

7261 ccgtccacgg gtgtgctctg ccccccagca agcaaacccc cgttcctcct cccaggagga

7321 agaggacggt tcagctcact gagtccgttg ttaccactgc tttggcagag ctggccgcga

7381 agaccttcgg ccagtcagag ctgggctcgg actccggcgc agacctcgcc accccaactg

7441 agaccacaga ctcgggcccc ctcattgtgg atgacgcatc cgatgacgga tcttattcgt

7501 caatgcctcc actagagggg gagcccggtg acccggactt ggcatcagat tcttggtcca

7561 ctgttagcgg atcagaggac gtcgtgtgct gctcaatgtc gtactcatgg actggggcgc

7621 ttgtgacacc ttgcgcggct gaggaatcaa agctgccaat tagccccctg agcaattcac

7681 tcttacgcca tcacaacatg gtgtatgcca cgaccacccg ttctgccgta acccggcaga

7741 agaaggtgac ctttgaccgc ctgcaggtgg tagacaacca ttacaatgat gtactcaagg

7801 agatcaaagc acgagcatcc agagtgaagg cacgcttgct taccacggag gaagcttgcg

7861 acctgacgcc cccccactct gccaaatcga aattcggcta tggggcgaag gatgttcgta

7921 gctattcccg caaggccatt aaccacatca actccgtgtg ggaggacttg ctggatgaca

7981 acaatactcc aataccaaca acaatcatgg ccaaaaatga ggttttcgtt gtgaacgcag

8041 cgaagggagg tcggaagcct gcccgcctga tcgtgtatcc ggatcttggg gttcgggttt

8101 gcgagaagag ggcgcttcac gacgtcgtca aacagctacc tgaggccgtg atgggagccg

8161 cttacggctt ccagtactcc ccagcgcagc gggtagattt tcttctgact gcttggaagt

8221 caaagaagaa tcctatgggg ttctcttatg acacccgctg ctttgactcc actgtaaccg

8281 aaaaggacat cagagccgag gaagaggtct atcagtgttg tgacctggag cccgaagccc

8341 gcaaggttat tgccgccctc acagagagac tctacgtggg cggccccatg tataacagca

8401 agggagacct gtgtgggtat cggagatgcc gtgcaagcgg cgtctacacc accagcttcg

8461 gaaacacact gacgtgctat ctcaaagcta cagccgctat tagggcggcc gggctgagag

8521 actgcactat gctggtttgc ggcgacgact tagtcgtcat cgctgagagc gacggtgttg

8581 aggaggataa ccgaggtctc cgagccttcg cagaggctat gacgagatac tcggctcccc

8641 caggcgacgc cccgcaacca gcatatgacc tggaactaat aacatcatgt tcctccaacg

8701 tctcagtcgc gcacgacgcg acgggtaaga gggtatacta cctgacccga gaccctgaga

8761 caccccttggc gcgagccgca tgggagacag tccgacacac tccagtcaat tcctggttgg

8821 gaaacatcat agtctacgcc cccacaatat gggtgcgcat ggtattgatg acccactttt

8881 tctcaatact ccaaagccag gaagcccttg agaaagcact cgacttcgat atgtacggag

8941 tcacttactc tatcactccg ctggatctac cggcaatcat tcaaagactc catggcttga

9001 gcgcatttac actgcacgga tactctccac acgaactcaa tcgggtgtct ggaagcctca

9061 ggaaacttgg ggtacccccca ttgagagcgt ggagacatcg ggcccgagca gtccgcgcca

9121 agcttattgc ccagggaggt aaggccaaaa tctgtggcat atacctcttt aactgggcag

9181 taaaaaccaa gcttaaactc actccattgc ctgccgctgc caaactcgat ttatcgggtt

9241 ggttcacggt gggcgccggc gggggagaca tttatcacag catgtctcat gcccgacccc

9301 gctatttact cctgtgccta ctcctacttt cagtaggggt aggcattttc ctgctgcctg

9361 ctcggtaggc agcttaacac tccgacctta gggtccctgt tttttttttt tttttttttt

9421 tttttttttt tttttttttt tttttttttt tttttttttt tttttttttt tttttttttt

9481 tcctttttcc ttcttttctc ttcttaatcc ttctttcttg gtggctccat cttagcccta

9541 gtcacggcta gctgtgaaag gtccgtgagc cgcatgactg cagagagtgc tgaaactggc

9601 ctctctgcag atcatgt
