# Supplementary material for: Longer Poly(U) Stretches in the 3′UTR Are Essential for Replication of the Hepatitis C Virus Genotype 4a Clone in in vitro and in vivo
Source: Front Microbiol. 2021 Nov 25;12:764816. doi: 10.3389/fmicb.2021.764816 (PMC8656456; doi:10.3389/fmicb.2021.764816)
Supplement: Supplementary Figure 3 — (A) Comparison of HCV-G4 KM long and short nucleotide sequences and substituted nucleotides within coding region were shown. Nucleotide substitutions in coding region without amino acid changes are indicated by the red color. Other sequences were all conserved between HCV-G4 KM long and short. (B) Comparison of HCV-G4 KM long and short and nucleotide differences within the 3′UTR were shown. The translation stop codon is indicated with a red square, and positions of U-stretch and 3′X regions are indicated. [file Table_3.pdf]

A

Substitution without amino acid change

```
long    8881  TCTCAATACTCCAAAGCCAGGAAGCCCTTGAGAAAGCACTCGACTTCGATATGTACGGAG  8940
              ********************************************************** *
short   8881  TCTCAATACTCCAAAGCCAGGAAGCCCTTGAGAAAGCACTCGACTTCGATATGTACGGGG  8940

long    8941  TCACTTACTCTATCACTCCGCTGGATCTACCGGCAATCATTCAAAGACTCCATGGCTTGA  9000
              ************************************************************
short   8941  TCACTTACTCTATCACTCCGCTGGATCTACCGGCAATCATTCAAAGACTCCATGGCTTGA  9000

long    9001  GCGCATTTACACTGCACGGATACTCTCCACACGAACTCAATCGGGTGTCTGGAAGCCTCA  9060
              ************************************************************
short   9001  GCGCATTTACACTGCACGGATACTCTCCACACGAACTCAATCGGGTGTCTGGAAGCCTCA  9060

long    9061  GGAAACTTGGGGTACCCCCATTGAGAGCGTGGAGACATCGGGCCCGAGCAGTCCGCGCCA  9120
              ******************* ****************************************
short   9061  GGAAACTTGGGGTACCCCCGTTGAGAGCGTGGAGACATCGGGCCCGAGCAGTCCGCGCCA  9120
```

B

```
                    Stop                                          U stretch
long    9361  CTCGGTAGGCAGCTTAACACTCCGACCTTAGGGTCCCTGTTTTTTTTTTTTTTTTTTTTT  9420
              **************************************************
short   9361  CTCGGTAGGCAGCTTAACACTCCGACCTTAGGGTCCCTGTTTTTTTTTTT----------  9410

long    9421  TTTTTTTTTTTTTTTTTTTTTTTTTTTTTTTTTTTTTTTTTTTTTTTTTTTTTTTTTTTT  9480

short   9410  ------------------------------------------------------------  9410
                                                                  3'X region
long    9481  TCCTTTTTCCTTCTTTTCTCTTCTTAATCCTTCTTTCTTGGTGGCTCCATCTTAGCCCTA  9540
                **********************************************************
short   9411  --CTTTTTCCTTCTTTTCTCTTCTTAATCCTTCTTTCTTGGTGGCTCCATCTTAGCCCTA  9468

long    9541  GTCACGGCTAGCTGTGAAAGGTCCGTGAGCCGCATGACTGCAGAGAGTGCTGAAACTGGC  9600
              ************************************************************
short   9469  GTCACGGCTAGCTGTGAAAGGTCCGTGAGCCGCATGACTGCAGAGAGTGCTGAAACTGGC  9528

long   9601  CTCTCTGCAGATCATGT  9617
             *****************
short  9529  CTCTCTGCAGATCATGT  9545
```

Suppl. Fig.3
